# The rise and fall of glucocorticoids and immunosuppressants in idiopathic pulmonary fibrosis: a historical paradigm shift driven by mechanistic understanding

**DOI:** 10.3389/fimmu.2026.1830573

**Published:** 2026-05-19

**Authors:** Bing Wen, Xue Wu, Xiaosheng Ma, Dan Li, Li Qian

**Affiliations:** 1 The First Clinical Medical College of Shanxi Medical University, Taiyuan, China; 2 Department of Geriatrics, Taiyuan Second People's Hospital, Taiyuan, China; 3 Department of Respiratory and Critical Care Medicine, Taiyuan Central Hospital, The Ninth Clinical Medical College of Shanxi Medical University, Taiyuan, China; 4 The First Hospital of Shanxi Medical University, Taiyuan, China

**Keywords:** glucocorticoids, idiopathic pulmonary fibrosis, immunosuppressive agents, macrophages, mechanical stress, myofibroblasts

## Abstract

The therapeutic strategy for Idiopathic Pulmonary Fibrosis (IPF) has undergone a fundamental transformation over recent decades. Combination therapy with glucocorticoids and immunosuppressants, particularly the “triple therapy” regimen including corticosteroids, azathioprine, and an antioxidant, has shifted from being the standard of care to being completely abandoned. This article reviews the key evidence-based milestones in this historical shift and, grounded in a modern understanding of the disease’s nature, delves into the pathophysiological basis for the ineffectiveness and even harm of glucocorticoids and immunosuppressants in IPF. The core argument posits that IPF is a disorder of immune microenvironment dysregulation, initiated by persistent alveolar epithelial injury, driven by pro-fibrotic phenotype macrophages, and ultimately resulting in myofibroblast activation and aberrant extracellular matrix deposition. Traditional glucocorticoids and immunosuppressants, whose primary target cells are lymphocytes, are misaligned with the core immune drivers (macrophages) and effector cells (myofibroblasts) of IPF. Consequently, they not only fail to reverse the fibrotic process but may also potentially promote fibrogenesis by enhancing transforming growth factor-beta (TGF-β) signaling pathways, inducing factors like CTGF, and they carry significant safety risks. Current treatment for IPF has fully transitioned into an era centered on antifibrotic therapy.

Idiopathic Pulmonary Fibrosis (IPF) is a chronic, progressive, fibrotic interstitial lung disease of unknown cause, characterized by the formation of fibrotic scars in the lungs leading to Usual Interstitial Pneumonia (UIP). It manifests clinically as progressive dyspnea, dry cough, and an irreversible decline in lung function, with a poor prognosis and a median survival of only 2 to 3 years (1). For a long time, IPF was considered an “inflammation-driven” disease, and therefore anti-inflammatory and immunosuppressive therapies were widely adopted (2). However, with a deeper understanding of its pathogenesis, the therapeutic approach to IPF has undergone a fundamental shift. This article aims to systematically review the changing historical role of glucocorticoids and immunosuppressants in the treatment of IPF and explore the intrinsic mechanisms underlying their failure from a modern pathophysiological perspective.

## 1 Historical evolution of glucocorticoids/immunosuppressant therapy in IPF

In the 1980s, based on the “inflammation-injury-repair” hypothesis, IPF was believed to originate from persistent alveolitis of unknown cause (3). Supported by this theory, in 1999, the American Thoracic Society recommended a “triple therapy” regimen centered on “anti-inflammation-immunosuppression-antioxidation,” namely prednisone (glucocorticoid) + azathioprine (immunosuppressant) + N-acetylcysteine (antioxidant), which was long considered the standard treatment for IPF (2, 4). From a pharmacological perspective, glucocorticoids (e.g., prednisone) exert broad anti-inflammatory and immunosuppressive effects through genomic and non-genomic mechanisms: the hormone-receptor complex enters the nucleus to regulate the transcription of inflammation-related genes, inhibiting inflammatory cell activity and the production of inflammatory factors; they can also rapidly reduce vascular permeability and exudation (5, 6). Azathioprine, as an antimetabolite immunosuppressant, has its metabolite, 6-thioguanine nucleotide, incorporated into DNA, inducing T-cell apoptosis and thereby inhibiting lymphocyte proliferation (7). This combination was thought at the time to effectively control alveolar inflammation and delay disease progression.

However, with the accumulation of evidence-based medicine, the role of traditional immunosuppressive regimens in IPF began to be challenged. As early as 1991, Raghu et al. conducted a randomized double-blind placebo-controlled clinical trial evaluating azathioprine combined with prednisone for IPF. The study enrolled 27 treatment-naive IPF patients, randomly assigned to receive prednisone plus azathioprine (n=14) or prednisone plus placebo (n=13). Results showed that at one year of treatment, there were no statistically significant differences between the two groups in changes in pulmonary function parameters (alveolar-arterial oxygen gradient, FVC, DLCO). Although the azathioprine group showed a trend towards improved survival over the 9-year follow-up period (mortality 43% vs. 77%), Cox regression analysis showed an unadjusted hazard ratio (HR 0.48, 95% CI 0.17-1.38) that did not reach statistical significance; marginal significance (P = 0.05) was only achieved after age adjustment (4). In 2002, Cottin, in a review published in Presse Médicale, explicitly stated: “Recent studies employing stricter diagnostic criteria have failed to provide convincing evidence that glucocorticoids or immunosuppressants (cyclophosphamide, azathioprine) are effective in IPF” (8). The same year, a systematic review by Davies et al. also noted that while some early, methodologically weaker studies suggested potential benefit from immunosuppressants, these studies often included other pathological types with better responses to immunosuppressive therapy (such as Nonspecific Interstitial Pneumonia(NSIP)); however, “high-quality trials evaluating modern diagnostic criteria have shown disappointing results with interventions like colchicine and azathioprine” (9). Clinicians also explored various other immunosuppressants for IPF, but results were uniformly unsatisfactory: 1) Exploratory use of Cyclophosphamide: As an alkylating agent immunosuppressant, cyclophosphamide inhibits cell proliferation by crosslinking DNA. It was used in combination with prednisone in some IPF patients (10). However, the lack of high-quality randomized controlled trials confirming its efficacy, coupled with significant adverse effects like bone marrow suppression and infection, prevented its widespread adoption in clinical practice. 2) Attempts with other Immunosuppressants: Drugs such as methotrexate, colchicine, penicillamine, and cyclosporine were also tried in combination with prednisone for IPF, but all failed to demonstrate success due to lack of efficacy or poor tolerability (10). These experiences suggested that non-specific immunosuppression was unlikely to alter the disease course of IPF.

Entering the 21st century, with a deeper understanding of IPF pathogenesis, researchers began exploring more targeted immunomodulators. In 2004, Ganesh Raghu et al. conducted a randomized controlled trial of interferon gamma-1b for IPF (11). Interferon gamma-1b, a recombinant cytokine, exerts immunomodulatory effects by activating macrophages and enhancing natural killer cell activity, theoretically potentially improving IPF prognosis through anti-fibrotic mechanisms (12, 13). However, the study enrolled 330 IPF patients unresponsive to corticosteroid therapy and found that interferon gamma-1b failed to significantly improve progression-free survival, pulmonary function, or quality of life; although there was a trend towards reduced mortality in the interferon group (10% vs. 17%, P = 0.08), it did not reach statistical significance or. In 2008, the same team attempted etanercept, a tumor necrosis factor-alpha (TNF-α) inhibitor. Etanercept, a recombinant fusion protein, blocks TNF-α biological activity by competitively binding to it, thereby inhibiting inflammatory responses. A randomized controlled trial in 88 patients with clinically progressive IPF showed no significant difference in the primary endpoint between the etanercept and placebo groups (14).

Concurrently, exploration of traditional immunosuppressants in IPF continued. A retrospective study published in 2011 included 10 IPF patients treated with mycophenolate mofetil (2g/day) for 12 months. Results showed no significant improvement in forced vital capacity (FVC), total lung capacity (TLC), diffusing capacity for carbon monoxide (DLCO), or 6-minute walk distance. However, high-resolution CT assessment revealed significant worsening in both overall disease extent and ground-glass opacity extent (P values 0.002 and 0.02, respectively), suggesting that while mycophenolate mofetil had an acceptable safety profile, it failed to demonstrate any therapeutic benefit (15).

These studies further confirmed that targeted intervention against a single inflammatory pathway was insufficient to reverse the IPF process. Notably, although cyclophosphamide, mycophenolate mofetil, and methotrexate are traditional broad-spectrum immunosuppressants, interferon gamma-1b is an immune agonist, and etanercept is an anti-TNF-α inhibitor—three vastly different mechanisms of action—their common significance lies in the fact that neither traditional immunosuppression, nor directed immunomodulation (interferon gamma), nor targeted anti-inflammatory therapy (etanercept) demonstrated clear efficacy in IPF. This set the crucial stage for the landmark findings of the PANTHER-IPF study: the pathological nature of IPF might be fundamentally different from traditional inflammatory diseases.

In 2012, Ganesh Raghu et al. published the PANTHER-IPF study (Prednisone, Azathioprine, and N-Acetylcysteine: A Study That Evaluates Response in Idiopathic Pulmonary Fibrosis), a multicenter, randomized, double-blind, placebo-controlled clinical trial designed to assess the efficacy and safety of triple therapy (prednisone + azathioprine + N-acetylcysteine) in IPF patients (16). The study was initially planned for 60 weeks of follow-up, but during an interim analysis, the Data and Safety Monitoring Board found that the triple therapy group exhibited significant harm compared to the placebo group: increased mortality (11% vs. 1%), increased hospitalization rates (29% vs. 8%), and increased serious adverse events (31% vs. 9%), leading to premature termination of the study (16). This high-quality evidence provided the first clear indication that triple therapy was not only ineffective but also definitively harmful for IPF patients, marking a fundamental turning point in the IPF treatment paradigm (16, 17). Subsequently, the 2015 ATS/ERS/JRS/ALAT guidelines recommended that clinicians should not use the combination therapy of N-acetylcysteine, azathioprine, and prednisone in patients with IPF (18).

The PANTHER-IPF study is a landmark in IPF treatment. However, due to clinical inertia (The inertial dependence of clinical decisions on established treatment models) in treatment approaches, or confounding factors like diagnostic uncertainty or the inflammatory response during acute exacerbations, some clinicians still resorted to using Glucocorticoids and Immunosuppressants in IPF patients. Therefore, even after PANTHER-IPF, numerous researchers continued to demonstrate the ineffectiveness of traditional immunosuppressive regimens in IPF. A randomized controlled trial from Japan published in 2015 compared cyclosporine A combined with low-dose corticosteroids versus cyclophosphamide combined with low-dose corticosteroids for IPF. The study enrolled 99 patients with a definitive diagnosis of IPF and followed them for 48 weeks. It found no significant difference between the two groups in the primary endpoint of change in FVC (-0.078 L and -0.087 L, respectively), and the incidence of adverse events was also similar (19). Although the study was underpowered to draw definitive conclusions due to its small sample size, it further supported the view that calcineurin inhibitors also fail to improve outcomes in IPF.

Even in the specific clinical context of acute exacerbation of IPF, the benefit of immunosuppressive therapy has not been confirmed. A study by Papiris et al. in 2015 suggested that exposure to immunosuppressants prior to an acute exacerbation of IPF was negatively correlated with patient survival. The researchers thus proposed that “avoiding corticosteroid use might be beneficial for the natural course of IPF, even in the most severe event of acute exacerbation” (20). The Effect of Cyclophosphamide Added to Glucocorticoids in Acute Exacerbation of Idiopathic Pulmonary Fibrosis(EXAFIP study), published in 2022, a randomized, double-blind, placebo-controlled phase III trial, aimed to evaluate the efficacy of adding cyclophosphamide to glucocorticoids for treating acute exacerbations of IPF. Results showed no significant difference in the primary endpoint of survival between the cyclophosphamide and placebo groups (21). Collectively, this evidence points to a single conclusion: whether in the stable phase or during acute exacerbations, the use of glucocorticoids and immunosuppressants has failed to demonstrate clear efficacy in IPF, with some regimens causing definite harm.

Subsequently, the updated 2022 guidelines explicitly state that the cornerstone of IPF treatment is antifibrotic drugs (such as nintedanib and pirfenidone), completely omitting any mention of glucocorticoids and immunosuppressants in the management of IPF (22), marking a complete shift in the IPF treatment philosophy. The 2025 ERS/ATS statement, while primarily focused on updating the international multidisciplinary classification of interstitial pneumonias, still points out: the core treatment for IPF remains antifibrotic therapy, which can improve prognosis. Routine use of corticosteroids/immunosuppressants is not recommended. It specifically notes that IPF patients with short telomere length (<10th percentile) may derive less benefit and face higher harm from immunosuppressants, and their use should only be considered cautiously after excluding other comorbidities (such as autoimmune features) and following multidisciplinary evaluation (23).

**Figure 1:**
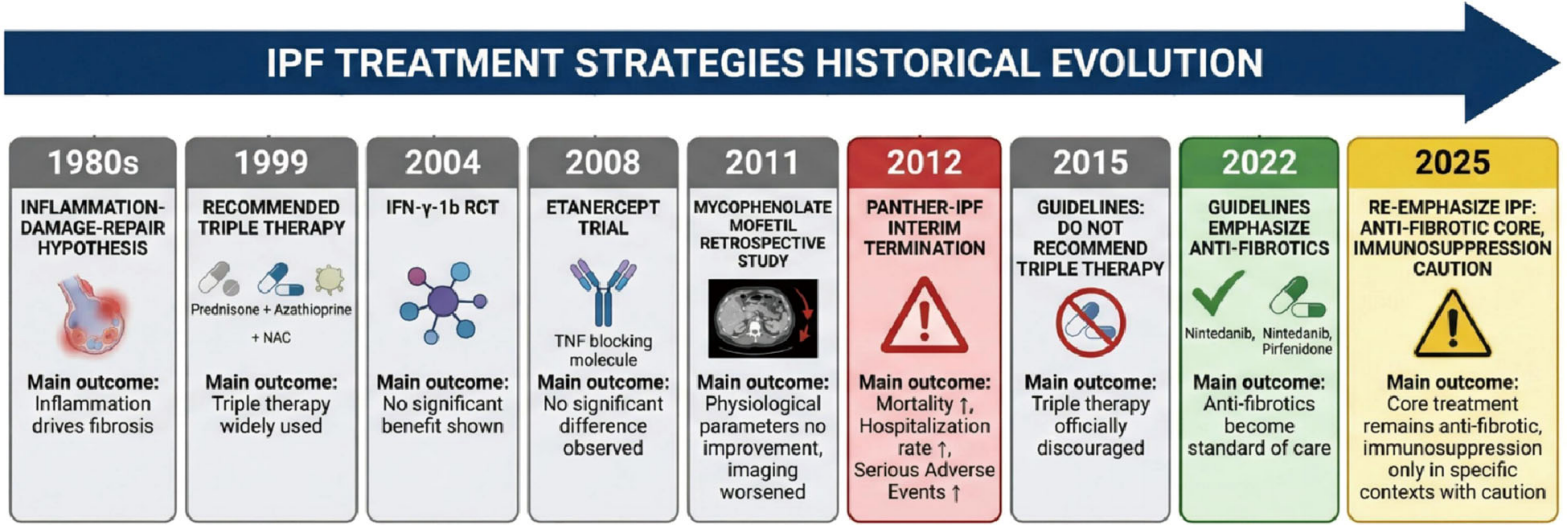
Historical evolution of treatment paradigms for idiopathic pulmonary fibrosis.

It is noteworthy that the efficacy of immunosuppressive therapy in UIP-type interstitial pneumonia depends on the underlying disease context. A review published in 2025 systematically examined the evidence for immunosuppressive therapy in UIP associated with autoimmune rheumatic diseases. It noted that in systemic sclerosis-associated Interstitial Lung Disease (ILD), patients with a UIP pattern showed a non-significant trend towards worsening under immunosuppressive therapy. In contrast, in rheumatoid arthritis-associated ILD and interstitial pneumonia with autoimmune features (IPAF), immunosuppressive therapy demonstrated positive effects (24). In systemic sclerosis-associated ILD (SSc-ILD), the Scleroderma Lung Study II (SLS-II), a randomized controlled trial comparing mycophenolate mofetil (MMF) with oral cyclophosphamide (CYC), demonstrated that both agents significantly improved lung function, dyspnea, and radiographic outcomes in SSc-ILD patients—including those with a UIP pattern—after 24 months of treatment (25). In rheumatoid arthritis-associated ILD (RA-ILD), a large real-world, multi-center study including 212 patients demonstrated that immunosuppression with mycophenolate mofetil, azathioprine, or rituximab was associated with an improved trajectory in FVC and diffusing capacity for carbon monoxide (DLCO) compared with pre-treatment pulmonary function trajectory; notably, the presence of a UIP pattern did not significantly impact treatment response (26).This comparison further emphasizes that immunosuppressants should be avoided in classical IPF, whereas they may be beneficial in fibrotic ILDs associated with autoimmune diseases. The major milestones in this paradigm shift are illustrated in **Figure 1**.

## 2 Mechanisms underlying the inappropriateness of glucocorticoids/immunosuppressants in IPF

Recent research indicates that IPF is, in essence, still an inflammation-driven disease. However, its inflammatory mechanism is fundamentally distinct from traditional pathogen infection, autoimmune inflammation, or lymphocyte-dominated inflammation. The current consensus conceptualizes IPF’s initiation model as an “inflammatory trigger model” involving “recurrent alveolar epithelial injury—abnormal repair—fibrosis” (27). The cells driving the progressive fibrosis are pro-fibrotic phenotype macrophages, derived from recruited peripheral monocytes (28). These macrophages create a pro-fibrotic microenvironment while simultaneously promoting the transformation of fibroblasts into myofibroblasts. Ultimately, activated myofibroblasts are the core effector cells responsible for collagen secretion and aberrant extracellular matrix deposition (29). Thus, the pathogenesis of IPF can be summarized into three phases: the “initiation phase,” the “driving phase,” and the “effector phase.”

### 2.1 The initiation phase of IPF and the closure of the therapeutic window

In healthy individuals, the lung is constantly exposed to the external environment, undergoing continuous cycles of injury and repair. This process maintains a dynamic balance. But in IPF patients, this “injury-repair” balance is disrupted. Repeated injury to alveolar epithelial cells (AECs) leads to chronic inflammation, generating chemokines, cytokines, and growth factors. The production ratios of these factors become imbalanced (29), subsequently recruiting large numbers of monocytes/macrophages into the lung. These cells transform into pro-fibrotic phenotype macrophages, establishing a pro-fibrotic microenvironment within the lung, ultimately resulting in pulmonary fibrosis (28). Of course, lymphocytes are also among the immune cells recruited early on. A prospective study by Sahajal Dhooria et al. published in 2025, involving 150 patients with non-IPF interstitial lung disease, showed that a higher bronchoalveolar lavage lymphocyte percentage (BLP) and a shorter duration of ILD-related symptoms (such as dyspnea, cough) were independent predictors of early glucocorticoid response. Notably, patients with BLP ≥ 20% and symptoms lasting less than 6 months exhibited significantly better responses to glucocorticoid therapy (30). This finding suggests that in other interstitial lung diseases characterized by lymphocytic infiltration (e.g., hypersensitivity pneumonitis, non-specific interstitial pneumonia), corticosteroid therapy holds clear value. However, in the progression of IPF, lymphocytes are not the core drivers of fibrosis. Considering the clinical presentation, most IPF patients are already in an advanced fibrotic stage at the time of diagnosis, meaning the disease has progressed from the “initiation phase” to the “driving phase” or even the “effector phase,” making the application of glucocorticoids/immunosuppressants miss the therapeutic window. Furthermore, even if applied earlier, these agents, which primarily target lymphocytes, are unlikely to halt the fibrotic process driven by macrophages.

### 2.2 The primary functional cell in the driving phase is the pro-fibrotic phenotype macrophage

Recent single-cell studies have clearly revealed that during the fibrotic driving phase of IPF, the predominant immune cell is the macrophage, not the traditionally emphasized lymphocyte.

Firstly, regarding cellular composition, fibrotic foci are enriched with specific pro-fibrotic macrophage subsets. In 2019, Christina Morse et al., using single-cell sequencing technology, identified three discrete macrophage subsets in normal and fibrotic lung tissue: one expressing monocyte markers, one with high expression of FABP4 and INHBA (FABP4^hi^), and another with high expression of SPP1 and MERTK (SPP1^hi^). Notably, the SPP1^hi^ macrophages were significantly enriched in the fibrotic lesions of IPF patients, suggesting they are a key cell type driving fibrotic progression. They pointed to targeting the proliferation of pro-fibrotic phenotype macrophages as a potential therapeutic strategy for IPF (31).

Further research has elucidated how these macrophages are recruited, polarized, and execute their pro-fibrotic functions, forming a complete driving cascade:

Recruitment: Injured alveolar epithelial cells and activated interstitial cells release various factors (e.g., TGF-β, IL-1β, CCL2, CCL12) that recruit peripheral blood monocytes into the lung tissue via signaling axes like CCR2, where they differentiate into monocyte-derived macrophages. Animal studies have confirmed that blocking CCL2 signaling significantly reduces macrophage recruitment and attenuates pulmonary fibrosis (32).

Polarization: Recruited monocyte-macrophages undergo polarization induced by local microenvironmental signals (e.g., IL-4, IL-13, TGF-β), transitioning towards a pro-fibrotic phenotype (such as M2-like, SPP1^hi^ macrophages) (33–35). These polarized macrophages highly express various pro-fibrotic mediators, including TGF-β1, PDGF, and CTGF, thereby establishing a pro-fibrotic immune microenvironment locally (35).

Activation and Effector Function: Activated pro-fibrotic phenotype macrophages, through paracrine signaling and direct interaction with fibroblasts, drive the transformation of fibroblasts into myofibroblasts and promote the aberrant deposition of extracellular matrix (34–36). Concurrently, TGF-β1 expressed by these macrophages activates Smad signaling pathways, further amplifying the fibrotic effect (32).

Thus, the recruitment, polarization, and activation of macrophages constitute the core driving force propelling IPF from “initiation” to “progression.”

In stark contrast, the role of lymphocytes in this driving phase appears relatively limited.

A 2023 single-cell analysis study by Avraham Unterman et al. demonstrated that the proportion of CD14^+^CD163^-^HLA-DR^low^ monocytes in the peripheral blood of IPF patients increases significantly with disease progression, while lymphocyte counts decrease (37). In 2024, Theodoros Karampitsakos et al. showed that within pulmonary fibrotic lesions, specific pro-fibrotic macrophage subsets like SPP1^hi^ are enriched. Furthermore, in IPF patients with high expression of genes associated with these pro-fibrotic macrophages, T-cell co-stimulatory genes such as CD28 and ICOS were significantly downregulated (38). These pieces of evidence collectively point to a conclusion: during the progressive phase of IPF, the immune microenvironment undergoes a shift from lymphocyte predominance towards macrophage predominance. however, This does not imply that the role of lymphocytes can be entirely overlooked. Multiple spatial transcriptomics studies have demonstrated the presence of abundant tertiary lymphoid structures (TLS) in the end-stage lung tissue of IPF patients; these structures contain T cells, B cells, plasma cells, and dendritic cells, and remain relatively stable throughout the disease course (39).These TLS may play a role in local antibody production and immune regulation. Nevertheless, current evidence indicates that the presence of TLS is not clearly associated with responsiveness to immunosuppressive therapy; on the contrary, traditional immunosuppressive regimens predominantly targeting lymphocytes have failed to improve prognosis in IPF. Therefore, the increased proportion of macrophages and the persistence of lymphocytic aggregates are not mutually contradictory—the latter may reflect disease-associated remodeling of the local immune microenvironment, yet its functional contribution is insufficient to confer clinical benefit from therapeutic strategies that target lymphocytes.

Based on the evidence above, it becomes clear that the driving phase of IPF involves the recruitment of various immune cells, including lymphocytes and monocytes/macrophages. However, as the disease progresses, the proportion of monocytes/macrophages gradually increases, while the lymphocyte proportion decreases. Glucocorticoids and immunosuppressants primarily target lymphocytes, but have minimal impact on the recruitment, polarization, activation, and function of macrophages—the core immune force driving fibrosis. This “therapeutic target mismatch” is the key mechanism underlying the failure of traditional immunosuppressive regimens in IPF.

### 2.3 The vicious cycle in the effector phase

The large number of pro-fibrotic phenotype macrophages recruited during the driving phase interact with various cells, producing abundant pro-fibrotic factors. This establishes a pro-fibrotic microenvironment within the lung, This enables epithelial cells to directly transform into myofibroblasts through epithelial-mesenchymal transition (EMT), that is, under the induction of signals such as TGF-β, epithelial cells lose their epithelial phenotype, acquire mesenchymal characteristics, and start secreting extracellular matrix (ECM). The deposition of ECM further increases mechanical stress, which in turn activates myofibroblasts and epithelial cells to produce even more ECM, ultimately forming a positive feedback vicious cycle of “ECM—mechanical stress—fibrosis” (40). Moreover, mechanical stress not only acts on myofibroblasts but also affects surrounding epithelial cells. Donia W Ahmed et al., using biomaterials to mimic the local matrix cross-linking characteristic of early fibrosis on isolated cells, found that this local cross-linking can increase mechanosensing, differentiation, and new protein deposition and remodeling in alveolar epithelial cells (40). glucocorticoids and immunosuppressants are incapable of acting on fibroblasts to inhibit myofibroblast activation and function, and certainly cannot mitigate the effects of mechanical stress.

However, it should be noted that the specific time span over which IPF progresses from the “initiation phase” through the “driving phase” to the “effector phase” exhibits considerable inter-individual variability. At the time of clinical diagnosis, the majority of IPF patients are already in the middle-to-late stages of the disease—that is, they have already progressed beyond the initiation phase. Limited longitudinal studies suggest that progression from the initiation phase to end-stage fibrosis typically takes 2 to 5 years, and that once the positive mechanofeedback loop is established during the effector phase, the fibrotic process accelerates markedly. More precise temporal quantification remains to be elucidated by further prospective cohort studies.

**Figure 2:**
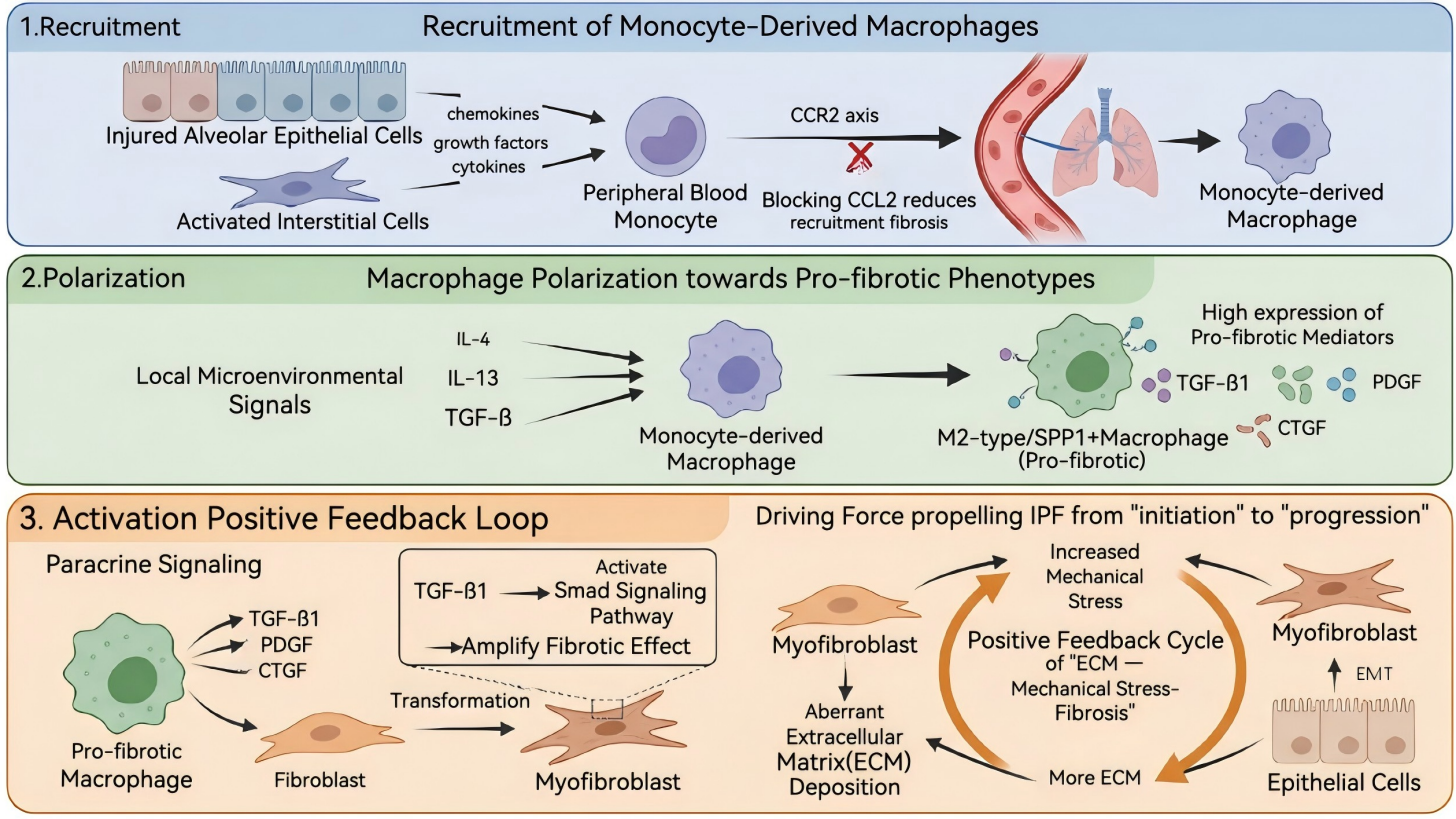
Cellular mechanisms driving fibrosis in idiopathic pulmonary fibrosis.

This pathological cascade, from epithelial injury to ECM deposition and mechanofeedback, is summarized in **Figure 2**.

### 2.4 Glucocorticoids may promote fibrosis and increase mortality

Julian T Schwartze et al., through *in vitro* and *in vivo* animal studies, discovered that glucocorticoids (GCs) (including dexamethasone, methylprednisolone, budesonide, and fluticasone) can: enhance TGF-β signaling via the ACVRL1/SMAD1/5/8 pathway, attenuate signaling via the TGFBR1/SMAD2/3 axis, induce the expression of the type III TGF-β receptor TGFBR3 (betaglycan), and ultimately synergize with TGF-β to drive the differentiation of primary lung fibroblasts into myofibroblasts (41). A nationwide cohort study from South Korea by Jung Hyun Nam et al. found that IPF patients receiving systemic corticosteroid therapy had significantly higher risks of mortality and acute exacerbation. The adjusted HRs for mortality in the corticosteroid group vs. the non-corticosteroid group were 1.63 and 1.38, respectively (42). The team led by Zhaoni Wang and Nanshan Zhong validated through animal experiments that dexamethasone can induce the expression of connective tissue growth factor (CTGF), promote fibronectin expression and fibroblast-to-myofibroblast differentiation, thereby exacerbating fibrosis (43). The single-cell analysis by Avraham Unterman et al. showed that the CD14^+^CD163^-^HLA-DR^low^ monocyte subset was proportionally higher in progressive IPF compared to stable IPF. High expression of this subset correlated with downregulation of T-cell co-stimulatory genes (CD28, ICOS, etc.), suggesting that HLA-DR^low^ monocytes might possess an immunosuppressive phenotype (37). Considering clinical observations that corticosteroid therapy may increase mortality risk in IPF patients, this suggests that for patients with advanced IPF who may already be in an immunosuppressive state, using corticosteroids for non-specific immunosuppression could further impair immune function, thereby accelerating the fibrotic process. Finally, the common side effects of glucocorticoids and immunosuppressants (such as increased infection risk, diabetes, gastrointestinal bleeding, osteoporosis, and metabolic disorders) are particularly pronounced in IPF patients. The increased risk of infection, in particular, may directly precipitate acute exacerbations, thereby significantly elevating patient mortality (8, 44).

## 3 Summary

In summary, IPF is a fibrotic disease initiated by alveolar epithelial injury, driven by macrophages, and sustained by myofibroblasts. The reasons for the failure of glucocorticoids/immunosuppressants in IPF can be attributed to: a “temporal mismatch” in the initiation phase; a “target mismatch” in the driving phase; and a “functional mismatch” in the effector phase. The withdrawal of glucocorticoids/immunosuppressants from the IPF therapeutic arsenal stands as a paradigm of evidence-based medicine triumphing over empirical experience.

Of course, in the development of therapies for pulmonary fibrosis, there have also been numerous failures. For example, the TGF-β signaling pathway, as a core driver of fibrosis, was once regarded as a highly promising target. However, the majority of clinical studies targeting TGF-β or its downstream effector molecules have been unsuccessful: the phase III ISABELA trial of ziritaxestat (an autotaxin inhibitor that suppresses LPA production to indirectly affect TGF-β signaling) was terminated early due to lack of efficacy (45).the phase III ZEPHYRUS-1 trial of pamrevlumab (a CTGF inhibitor, a downstream effector of TGF-β) failed to meet its primary endpoint (46). and the phase IIb trial of BG00011 (an αvβ6 integrin inhibitor that blocks TGF-β activation) was also discontinued because of poor efficacy (47).The repeated setbacks of TGF-β-targeted therapies may be attributable to the following reasons: 1) TGF-β exerts a wide range of physiological functions *in vivo* (such as immune regulation and tissue homeostasis), and systemic inhibition may lead to unacceptable toxic side effects; 2) the TGF-β signaling pathway possesses complex compensatory mechanisms and redundancy, such that blockade of a single node may be circumvented by alternative pathways. Clearly, the treatment of pulmonary fibrosis remains fraught with considerable difficulties.

The withdrawal of glucocorticoids/immunosuppressants from the historical stage of IPF treatment and the repeated failures of TGF-β-targeted drugs both suggest that the management of IPF can no longer rely on the “non-specific suppression” of a single inflammatory or fibrotic pathway. Instead, a precision medicine framework should be adopted, wherein patient stratification is performed based on biomarkers (such as IPF subtypes defined by immune cell characteristics, e.g., HLA-DRlowmonocytes and SPP1^+^ macrophages), thereby enabling the formulation of precise intervention strategies targeting core fibrotic effector cells and specific pathological immune cell populations. Considerable attempts in these areas have already been made. For instance, in the realm of targeting specific immune cell populations, precision interventions directed against pro-fibrotic macrophages and immunosuppressive monocytes—the core immunological driving force during the “driving phase”—are emerging as a research frontier. Single-cell sequencing studies have revealed that SPP1hi macrophages are highly enriched within fibrotic lesions of IPF, and that SPP1 secreted by these cells promotes M2 macrophage polarization through the JAK2/STAT3 pathway; targeting SPP1 can effectively attenuate the progression of fibrosis. Further research has demonstrated that the SPP1/CD44 axis participates in the progression of fibrosis by regulating aberrant lipid metabolism in alveolar macrophages, thereby providing a novel target for intervening in macrophage metabolic reprogramming (48) ;Moreover, the transcriptomic signature of CD14^+^CD163^-^HLA-DRlow monocytes has been confirmed to be significantly associated with mortality in IPF patients, and reversing the transcriptional program of this cell population holds promise as a novel precision therapeutic strategy (48). Admittedly, this line of investigation is still in its infancy and requires further intensive study. At the level of macrophage chemotaxis, the CCL2/CCR2 axis has been validated as a key pathway driving macrophage recruitment into the lung and M1 polarization; the delivery of siCCR2 using nucleic acid nanocarriers can effectively block macrophage accumulation and inhibit myofibroblast activation, demonstrating translational potential (49). In the near future, the treatment of IPF will undoubtedly transition from “slowing disease progression” toward a new era of “halting” or even “reversing” fibrosis.
